# Voces marginadas, voces recuperadas: dilemas del trabajo con archivos de la locura en América Latina

**DOI:** 10.1590/S0104-59702023000100022

**Published:** 2023-05-15

**Authors:** Nicolás Duffau

**Affiliations:** i Facultad de Humanidades y Ciencias de la Educación/Universidad de la República. Montevideo – Uruguay nicolasduffausoto@gmail.com

Desde la década de 1980, un grupo de investigadores de varios países latinoamericanos comenzó el arduo proceso de dotar de historicidad a los establecimientos de contención y reclusión de pacientes con afecciones psiquiátricas. Claramente influenciados por el impacto que generó la recepción de la obra de Michel Foucault (1967) y Roy Porter (1989) , por el fin de las dictaduras de seguridad nacional, los proyectos de reforma estatal de la salud pública pos consenso de Washington y los movimientos favorables a la desmanicomialización y la antipsiquiatría, investigadores de distintos países (pero especialmente de Argentina, Brasil y México) produjeron obras que problematizaron el rol histórico de los hospitales psiquiátricos, la construcción de campos de saberes expertos o la irrupción del psicoanálisis. Estas primeras investigaciones se desarrollaron a partir del análisis de documentación hasta entonces no explorada, como historias clínicas, archivos judiciales, escritos médicos e incluso testimonios de pacientes.

En los abordajes iniciáticos de las décadas de 1980 y 1990, los investigadores analizaban la documentación en forma casi instintiva, cumpliendo además con la (difícil) tarea patrimonial de conservar y garantizar la preservación de sus fuentes. Esos recorridos individuales se convirtieron en aportes colectivos, con marcos de análisis que actualmente se podrían considerar comunes, que, si bien no desconocen la especificidad de cada caso, se han concentrado en una problematización permanente de los documentos con los que se trabaja.

El libro coordinado por Teresa Ordorika Sacristán y Aída Alejandra Golcman (2021) es una reflexión coral que aporta a la renovación metodológica permanente en el campo de la investigación *psi* . Asimismo, reúne a un conjunto de investigadores que en las últimas dos décadas han producido textos fundamentales no sólo para la historia de la psiquiatría, la psicología o el psicoanálisis, sino también para la historia social y cultural de América Latina.

El libro se divide en cuatro secciones. La primera (“Expedientes clínicos”) está dedicada a los abordajes de historias clínicas y se conforma con los textos de Ana Teresa Venancio, Yonissa Marmitt Wadi, de las coordinadoras de todo el trabajo y de Andrés Ríos Molina. Los artículos exploran las posibilidades históricas de usar esos testimonios clínicos para descripciones colectivas/seriadas, así como para abordajes individuales que permiten operaciones biográficas. Se trata asimismo de un tipo de documento coproducido entre un mecanismo de poder (médico) y uno subalternizado (paciente), atravesado además por variables étnico-sociales, de género o clase. Todos los artículos que conforman esta sección plantean un debate, aún no resuelto por la historiografía, aquel que problematiza la tensión que se genera entre el análisis de un caso ejemplar y los intentos por desarrollar descripciones de carácter general.

Este mismo problema irresoluto también está presente en la segunda sección del libro (“Procesos judiciales”), que, con los trabajos de Cristina Sacristán y Martha Santillán, incorpora reflexiones sobre otro tipo documental: los expedientes judiciales. En este caso las dos autoras incorporan un nuevo elemento para seguir procesos de judicialización de enfermos psiquiátricos. Es decir, además de los peritajes médicos, ambas demuestran los distintos mecanismos ajenos al mundo médico-legal que incidieron en el desarrollo de una causa civil o penal y terminaron siendo centrales en el resultado final del proceso. Por eso, advierten que este tipo de documento sólo se puede analizar en contraste con otras fuentes. Es decir, el estudio de enfermos psiquiátricos que adquirieron cierta notoriedad pública no puede abrevar sólo en el análisis de las producciones desde la experticia, porque desde el momento que una situación salta, por ejemplo, a las páginas de la prensa, también lleva a un juego constante que (nuevamente) tensiona el caso particular, y el tratamiento sugerido o la posición garantista de un abogado, con las demandas de una sociedad que comenta, sigue el caso y en ocasiones termina incidiendo en una condena. Las dos primeras partes del libro demuestran de buen modo que no alcanza sólo con adoptar el punto de vista de la documentación, sino que hay que prestar atención a los modos en los cuales las sociedades aprecian la locura, opinan sobre distintas situaciones y también sienten cierta fascinación u horror.

Los textos de la tercera sección (“Novelas y periódicos”) también avanzan en esa relación entre las sociedades y las distintas formas de ver la locura. En esta parte del libro entramos a una dimensión que no sólo rescata el trabajo tradicional de investigación, sino que apuesta también a recuperar la experiencia en el proceso de investigación. Jonathan Ablard reevalúa su investigación en Argentina y cuestiona algunas de las conclusiones de su libro de 2008, en el que no planteó el uso de instituciones psiquiátricas para la represión en la dictadura civil militar (1976-1983) ( Ablard, 2008 ). A partir de un trabajo periodístico, el autor cuestiona su propio trabajo y avanza sobre el uso de las instituciones psiquiátricas para combatir a los enemigos políticos en las décadas de 1970 y 1980. Por su parte, José Antonio Maya toma como eje las narrativas de ficción como fuente para la historia de la psiquiatría y la literatura en México y explora la construcción de la locura como un fenómeno cultural. A tono con el trabajo de Ablard, Maya busca mostrar los resquicios de la actividad de investigación, contando su experiencia de trabajo. Para finalizar esa sección, Esteban Terán analiza las distintas nociones sobre los barbitúricos en México a mediados del siglo XX y evidencia los intersticios en los que se cruzan la mirada médica sobre el uso de drogas/estimulantes y las construcciones periodísticas que asociaron ciertos consumos con la enfermedad psiquiátrica.

La cuarta sección (“Cuestionarios, etnografías y entrevistas”), que cierra el libro, reúne dos experiencias de trabajo sobre la construcción de instrumentos para una descripción del campo *psi* actual. María Eugenia González reflexiona sobre las posibilidades de combinar el análisis cuantitativo y cualitativo para entender el campo profesional de los psicólogos en Argentina en un período reciente (2002-2012). Por último, Oliver Hernández Lara describe su experiencia etnográfica en dos instituciones hospitalarias mexicanas, y demuestra los resultados generados por la combinación entre la observación participante y la entrevista semiestructurada.

El libro constituye un aporte fundamental para la construcción del campo de investigaciones *psi* en América Latina, por la calidad de los trabajos individuales, pero también porque el conjunto llena un vacío metodológico e intenta sintetizar cuáles son los alcances y límites de los documentos para entender la locura. Asimismo, brinda pistas sobre qué reparos tenemos que tener al momento de trabajar con documentación que está inserta en tramas de poder (médico, jurídico, estatal) que alude a sectores subalternos. Es también un excelente ejemplo para entender los dispositivos que inciden en la conformación de un documento histórico y las precauciones a adoptar cuando intentamos comprender las conductas sociales a través de testimonios de este tipo. En suma, se trata de un excelente ejemplo de defensa del oficio de investigación porque recuerda que los documentos son una puerta de entrada a un problema histórico, pero son una huella que se convierte en fuentes de información sólo si los investigadores los interrogamos y problematizamos sus contextos de producción para transformar la función de mentira en confesión de verdad ( Le Goff, 1991 ).
